# A High-Throughput Screen for Tuberculosis Progression

**DOI:** 10.1371/journal.pone.0016779

**Published:** 2011-02-16

**Authors:** Ralph Carvalho, Jan de Sonneville, Oliver W. Stockhammer, Nigel D. L. Savage, Wouter J. Veneman, Tom H. M. Ottenhoff, Ron P. Dirks, Annemarie H. Meijer, Herman P. Spaink

**Affiliations:** 1 ZF-screens B.V., Leiden, The Netherlands; 2 Institute of Chemistry, Leiden University, Leiden, The Netherlands; 3 Institute of Biology, Leiden University, Leiden, The Netherlands; 4 Department of Immunohematology and Blood Transfusion, Leiden University Medical Centre, Leiden, The Netherlands; Charité-University Medicine Berlin, Germany

## Abstract

One-third of the world population is infected with *Mycobacterium tuberculosis* and multi-drug resistant strains are rapidly evolving. The noticeable absence of a whole organism high-throughput screening system for studying the progression of tuberculosis is fast becoming the bottleneck in tuberculosis research. We successfully developed such a system using the zebrafish *Mycobacterium marinum* infection model, which is a well-characterized model for tuberculosis progression with biomedical significance, mimicking hallmarks of human tuberculosis pathology. Importantly, we demonstrate the suitability of our system to directly study *M. tuberculosis*, showing for the first time that the human pathogen can propagate in this vertebrate model, resulting in similar early disease symptoms to those observed upon *M. marinum* infection. Our system is capable of screening for disease progression via robotic yolk injection of early embryos and visual flow screening of late-stage larvae. We also show that this system can reliably recapitulate the standard caudal vein injection method with a throughput level of 2,000 embryos per hour. We additionally demonstrate the possibility of studying signal transduction leading to disease progression using reverse genetics at high-throughput levels. Importantly, we use reference compounds to validate our system in the testing of molecules that prevent tuberculosis progression, making it highly suited for investigating novel anti-tuberculosis compounds *in vivo*.

## Introduction

Tuberculosis (TB) is an ancient chronic disease caused by *M. tuberculosis*. With one-third of the world population infected, the predominant outcome is a latent and persistent infection controlled by type I immune responses [1], [2], [3], [4]. An important characteristic of this infection is the formation of granulomatous lesions, consisting of clusters of infected macrophages and other immune cells [5], [6]. Paradoxically, the main purpose of the host macrophages, which *M. tuberculosis* infects and where it persists, is to clear bacterial infection [7], [8]. *M. tuberculosis* achieves persistent infection through rapid changes in its gene expression profile in order to counteract host cell biological and immune processes, such as antigen presentation, pro-inflammatory cytokine secretion and phagosome maturation [9].

The alarming rate of emergence of new drug resistant (MDR/XDR) *M. tuberculosis* strains isolated from patients, in particular HIV-infected individuals, is cause for global concern, and the race for more efficient vaccines, as well as novel antibiotics targeting either the pathogen or the host, has begun [3], [10], [11]. While *in vitro* models have shed light on processes that are central to the uptake and survival of the bacterium, they cannot recapitulate the full phenotype of latent *M. tuberculosis* infection. This has been partly circumvented through the use of non-human primate models, which develop a form of TB that exhibits many of the hallmarks of the human infection [12]. Other *in vivo* systems include the guinea pig model, used to validate anti-TB vaccines and drugs [13], and mouse models, which offer extensive arrays of genetic tools. However, neither rodent model fully recapitulates essential aspects of TB lesion progression in man, including granuloma formation and maturation [10], [14].

The low-cost and high clutch-size zebrafish (*D. rerio*) is, at the embryonal and larval stages, optically transparent, permitting visualization of pathogens and lesions in real time [15], as well as offering exciting possibilities for high-throughput imaging [16]. Zebrafish are also amenable to forward genetic screening, or reverse genetics techniques such as injection of morpholinos (inhibitory of mRNA translation) [17], [18]. An ectotherm, the zebrafish is one of the natural hosts of *M. marinum*, the closest relative of the *M. tuberculosis* complex [19]. Of crucial relevance, as shown by the pioneering work of the Ramakrishnan group, *M. marinum infection* of zebrafish closely mimics the mammalian TB pattern of infection, both in terms of bacterial numbers which increase rapidly in early infection, and of the formation of caseous granulomas that present characteristics typical of their human counterparts [20], [21], [22], [23].

The indirect study of human TB via the infection of the zebrafish embryo with *M. marinum* has already led to the clarification of many important processes in the life cycle of the infection, in particular those underlying the mechanisms of granuloma formation [22], [23], [24], [25], [26], [27]. The importance of studying mycobacterial infections at a whole organism level was highlighted in the report that induction of *mmp9* expression, enhancing macrophage recruitment to granulomas, was localized to epithelial cells near infected macrophages [26]. Another example of the use of zebrafish larvae to uncover a host-pathogen interaction relevant to human mycobacterial infection is the recent forward genetic screen by Tobin and Ramakrishnan, who mapped a hypersusceptibility mutation to the leukotriene biosynthesis gene, *lta4h*, and showed that heterozygosity at the *LTA4H* locus correlated with susceptibility of human populations to both TB and leprosy [28]. It is therefore clear that the zebrafish mycobacterial infection model is quickly becoming an attractive and advantageous alternative for analyzing granuloma and disease progression *in vivo*.

The common route of infecting zebrafish embryos with *M. marinum* is the injection of the pathogen into the caudal vein of the 1 day old embryo [23]. This method is labour-intensive and generally considered to be a low-throughput technique, leading to major bottlenecks in drug discovery, particularly in times of high-throughput technology. Since infection by immersion is not an effective alternative, we sought to achieve a reliable high-throughput automatic injection system, drastically reducing the man-hour requirement while vastly increasing the number of reproducibly infected embryos. Large quantities of similarly-injected/infected embryos would then allow testing of sizeable drug libraries for anti-bacterial activity targeting either the pathogen or the host itself.

Here we show that the automatic injector we developed provides a powerful and reliable high-throughput system for infecting embryos with *M. marinum*. We also show that we can couple the injector to a flow cytometer capable of sorting live multicellular organisms (Complex Object Parametric Analyzer and Sorter, COPAS) and rapidly test the efficacy of known anti-TB drugs in infected embryos. Finally, and importantly, we demonstrate that this system is ideally suited to test proliferation and tissue spreading of the human pathogen, *M. tuberculosis*.

## Results and Discussion

### Proof of principle of the yolk sac as an early-stage embryo injection site

**Figure 1:**
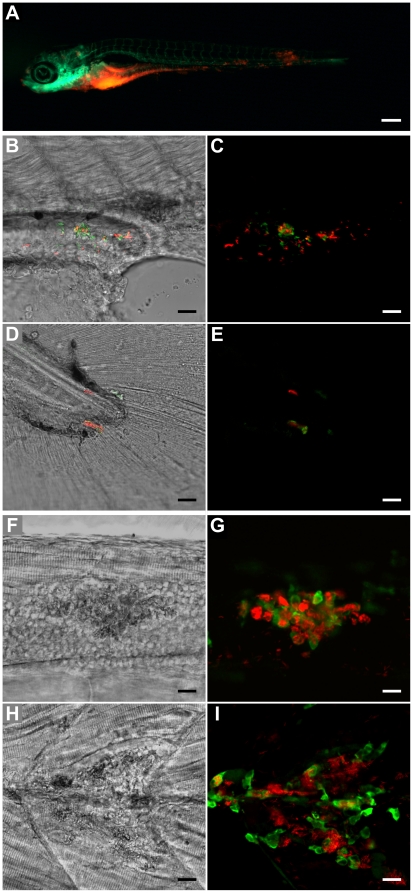
Outcome of *M. marinum* yolk sac injection of embryos between the 16- and the 512-cell stage. (**A**) 5 dpi fli1-egfp larva with gfp-labelled vasculature showing spread of bacteria (red) throughout the body (scale bar: 250 µm). (**B** and **D**) Bright-field/fluorescence overlay and (**C** and **E**) confocal z-stack of red-fluorescent bacteria showing activation of green-fluorescent *gags* at the (**B** and **C**) edge of the yolk extension and on the (**D** and **E**) tail of a 7 day-old larva (scale bar: 25 µm). (**F** and **H**) Bright-field confocal plane and (**G** and **I**) confocal z-stack of red-fluorescent bacteria co-localizing with green-fluorescent leukocytes detected by L-plastin immunostaining (scale bar: 25 µm). The lesions caused by the granulomas can be clearly seen in **F** and **H**.

**Figure 2:**
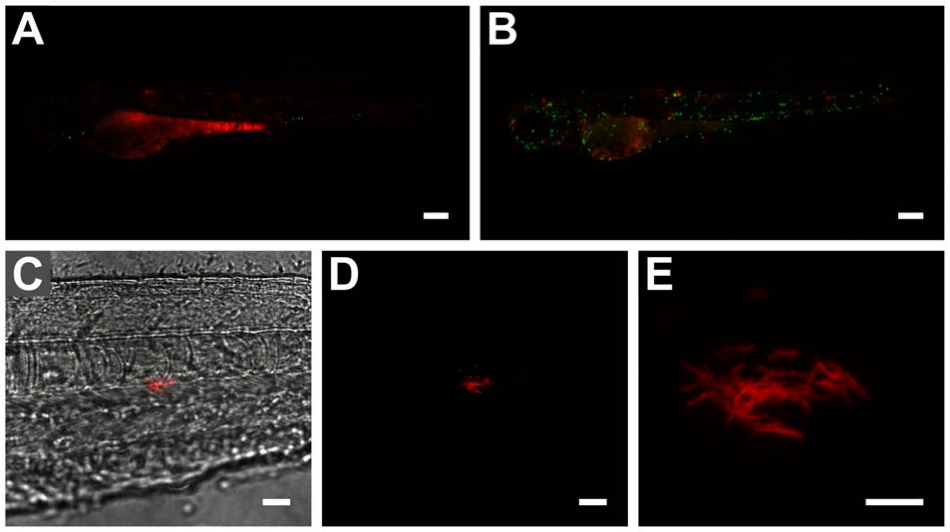
Effect of yolk sac co-injection of Pu.1 morpholino and *M. marinum* on bacterial localization and proliferation within embryos. (**A** and **B**) 3 day-old infected mpx-gfp transgenic embryos (**A**) with and (**B**) without Pu.1 morpholino (scale bar: 250 µm). Greater numbers of (extracellular) bacteria throughout body of morphant embryo seen in **A** contrast with lower amount of more localized (phagocytosed) bacteria seen in **B**. Very low number of mpx-gfp labelled neutrophils in **A** confirms Pu.1 morpholino effect. (**C**) Bright-field/fluorescence overlay and (**D**) confocal z-stack of *mag*49-GFP/mCherry bacteria in body of 2 dpi embryo (scale bar: 25 µm). Red-fluorescent bacteria form a cording structure adjacent to a few cells containing green-fluorescent (*mag*49-activated) bacteria. Lack of green fluorescence in cording bacteria indicates no phagocytosis by macrophages and extracellularity. (**E**) Close-up (digital zoom: 5.2) of cording structure formed by extracellular bacteria (scale bar: 10 µm; only red channel shown).

We first demonstrated that the injection of 20–40 *M. marinum* colony-forming units (CFUs) into the yolk sac of embryos at several early developmental stages (up to 1,024-cell stage) precisely mimics the infection obtained with the well-established caudal vein injection method. In our set-up, all injections were performed using polyvinylpyrrolidone as a polymer-based carrier for the bacteria, which showed several benefits: (1) restriction of early bacterial spread into the embryo, precluding developmental problems arising from the early injection stage; (2) higher concentration homogeneity; (3) clear visibility of injected inoculum as a spheroid (**Video S1**). Besides extensive bacterial growth within the yolk, we witnessed frequent formation of aggregates of infected cells outside the site of injection, namely in the head, body and tail of the larvae at 5 days post-infection (dpi) (**Figure 1A**). These aggregates were highly similar to those previously shown to represent initial stages of granuloma development [23]. No adverse developmental effects were seen in any of the conditions tested. Confirmation that yolk *M. marinum* injection resulted in granuloma formation was obtained through GFP-labelled *gag* (granuloma-activated gene) [23] activation at 7 dpi using an *M. marinum* strain also expressing mCherry (**Figure 1B–E**). Additionally, immunohistochemistry using L-plastin showed clear co-localization of *M. marinum* and leukocytes (**Figure 1F–I**). To functionally analyze the role of the immune system in the spreading and proliferation of mycobacteria after yolk injection, we co-injected a morpholino targeting *pu.1* [29] and *M. marinum* at the 1–2 cell stage. The results revealed the presence of extracellular *M. marinum* and increased bacterial proliferation in Pu.1 morphants (**Figure 2A and B**), consistent with previous data demonstrating that macrophages in zebrafish embryos restrict mycobacterial growth [25]. At 2 dpi, we observed cording structures in Pu.1 morphants, characteristic of extracellular mycobacteria [27], [28], in the tail region of infected embryos (**Figure 2C–E**). Using a *mag49* (macrophage-activated gene)-GFP [23] construct in mCherry-labelled bacteria, we were able to confirm their extracellular location through the lack of *mag*49-GFP expression, previously shown to be active only after phagocytosis by macrophages [23] (**Figure 2C and D**).

### High-throughput *M. Marinum* injection and drug screen

**Figure 3:**
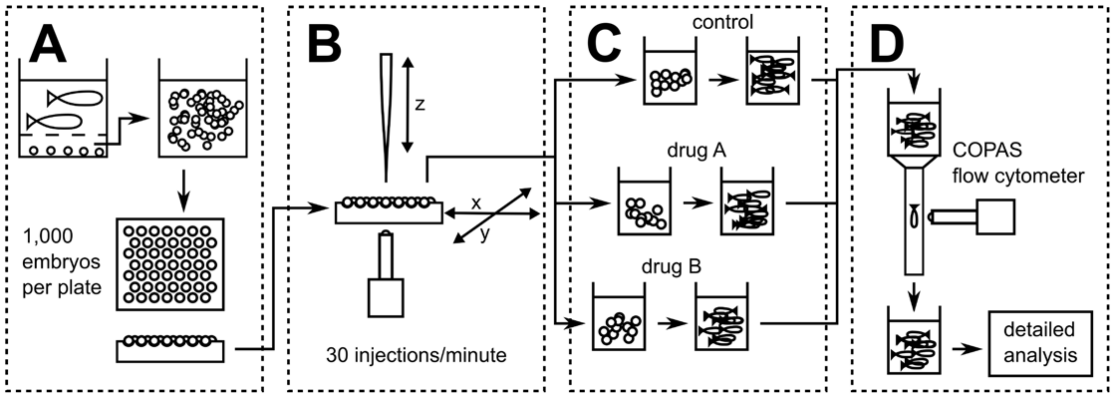
Pipeline of high-throughput infection of zebrafish embryos and subsequent drug testing. (**A**) After fertilization eggs are harvested, washed and distributed on injection plate. (**B**) Appropriate inoculum is injected in early stage embryos (up to 1,024-cell stage). (**C**) Injected embryos are dispensed into appropriate containers and drug screens take place between 3 and 6 dpi. (**D**) Groups of treated and untreated embryos are separately screened using COPAS during (when appropriate) and after drug exposure. Detailed optical analyses are performed on selected larvae.

**Figure 4:**
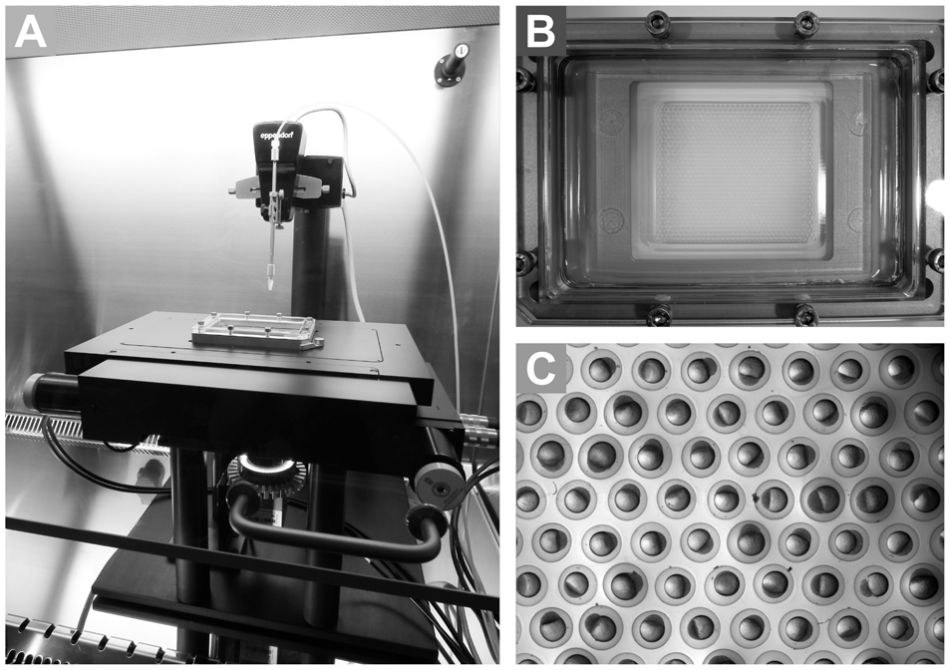
Pictures depicting automatic injector system. (**A**) the automatic injector system inside a laminar flow cabinet; (**B**) the embryo holder, showing the agarose grid within the steel support; (**C**) the embryo-filled grid, demonstrating the highly reproducible alignment of the embryos, with the cell mass resting to the side. Although size variation is observed, the embryos are always precisely in the centre of each well (the point of calibration for injection).

We subsequently developed an automatic injector system around the yolk injection concept (**Figure 3** and **4**). All tests performed demonstrated that this injector design, capable of 1,024 consecutive injections per run of 30 minutes, reproducibly reached a success rate of over 99% (sample in **Video S1**) and produced identical results to manual yolk injections of embryos. Importantly, embryos occupied the hemi-spherical wells of the agarose cast (**Figure 4B** **and S1**) in a centred and completely reproducible manner, with the cell mass always resting to the side (**Figure 4C**). No image recognition was thus required for the injections, unlike a previously reported design that operates at a throughput level of 25 consecutive injections per run of 2 minutes [30]. The choice of agarose as the casting material dramatically reduced light refraction, resulting in a better image during calibration, and helped maintain embryos humid and viable.

**Figure 5:**
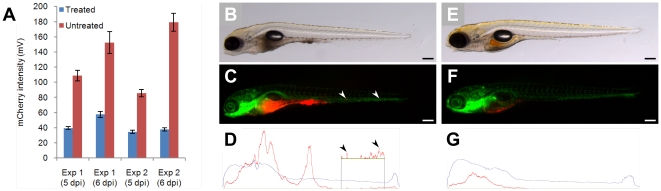
Automatic yolk sac injection of *M. marinum* and effect of treatment on infected larvae. (**A**) Effect of treatment on bacterial growth (measured by COPAS) in 5 and 6 day-old fli1-egfp larvae with gfp-labelled vasculature. Blue bars represent treated embryos, red bars represent untreated embryos. (**B–D**) Untreated versus (**E–G**) treated 5 day-old larvae, depicted whole in (**B** and **E**) bright-field and (**C** and **F**) fluorescent images, and (**D** and **G**) profiled by COPAS (scale bar: 250 µm). The localization of bacteria (red) in **C** and **F** correlates well with COPAS profile peaks in **D** and **G**, respectively (peaks in the tail region are shown enlarged in the inset; arrowheads depict two representative locations).

To demonstrate the applicability of our system to drug screens, two independent large sets of embryos were injected with *M. marinum* and treated with a combination of first-line anti-TB drugs (Rifampicin and Isoniazid). After 3 days, immediately prior to the start of the antibiotic treatment, embryos were run through the COPAS flow cytometry system to determine the total level of red fluorescence, representative of bacterial load. The embryos were subsequently split into two random groups, whereby one was subjected to the combinatorial treatment for 3 days. At 5 dpi, the average signal per larva in the untreated group was approximately 3-fold higher than that of the treated group, and this difference was even more pronounced (4-fold) at 6 dpi (**Figure 5A** and **Table S1**). These results attest both to the efficacy of the combinatorial drug treatment and to the ability of COPAS to correctly discriminate treated and untreated groups.

Epifluorescence and bright-field imaging revealed little to no red signal outside the yolk region of the treated larvae, which looked healthy and phenotypically normal. By contrast, untreated embryos displayed varying bacterial loads in the head, body and tail regions (**Figure 5B, C, E and F**). Additionally, the individual profiles generated by COPAS correctly indicated whether bacteria were present in the body of infected larva (**Figure 5D and G**). L-plastin immunostaining further confirmed co-localization of *M. marinum* and leukocytes in the body of the untreated larvae (**Figure 1F–I**).

### High-throughput *M. tuberculosis* injection and drug screen

It is clear that much can be learned about TB from the study of *M. marinum* infections in zebrafish, and the use of this pathogen offers practical advantages when compared to *M. tuberculosis*, such as lower biosafety restrictions and faster growth rate. That notwithstanding, it was of interest to study the human pathogen, *M. tuberculosis*, directly in zebrafish. Using our system, we overcame all technical difficulties of manually injecting a BSL-3 pathogen into zebrafish embryos. Two independent sets of embryos were injected with *M. tuberculosis*, and at 3 dpi were split into treated (combinatorial Rifampicin and Isoniazid treatment) and untreated groups. To support growth of *M. tuberculosis*, embryos were maintained at a higher temperature (34°C) than in *M. marinum* infections (28°C).

**Figure 6:**
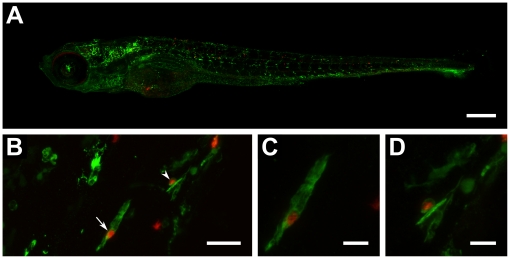
Automatic yolk sac injection of *M. tuberculosis* and effect of treatment on infected larvae. (**A**) Confocal z-stack (8×2 stitching) of a 6 day-old whole larva (fli1-egfp with gfp-labelled vasculature) showing spread of bacteria (red) throughout the body (scale bar: 250 µm). (**B**) Confocal z-stack of red-fluorescent bacteria co-localizing with green-fluorescent leukocytes detected by L-plastin immunostaining (scale bar: 25 µm). (**C**) Close-up (digital zoom: 4.2) of bacteria-containing leukocyte depicted in **B** by straight arrow (scale bar: 10 µm). (**D**) Close-up (digital zoom: 4.3) of bacteria-containing leukocyte depicted in **B** by arrowhead (scale bar: 10 µm).

Confocal imaging of fixed infected larvae revealed the presence of *M. tuberculosis* in their bodies after 5 dpi, indicating that the bacteria survived and were transported outside the injected area by macrophages, and that zebrafish larvae survive exposure to this pathogen (**Figure 6A**). There was a highly significant correlation (p = 0.0004) between *M. tuberculosis* presence in the larvae and the absence of treatment (**Figure S2**). Supporting the survival of *M. tuberculosis* in zebrafish, plating of lysates from 5 and 6 dpi untreated larvae resulted in growth of *M. tuberculosis* colonies. Noteworthy, treated larvae did not yield any colonies, implying that the bacteria were eliminated during treatment.

L-plastin immunostaining showed leukocytes clustering around infected regions throughout the body, suggesting the formation of granuloma-like aggregates similar to those observed in *M. marinum* infections (**Figure 6B–D**). Leukocytes in these aggregates showed intracellular fluorescence of *M. tuberculosis* bacteria (**Figure 6C and D**). In addition, we also observed bacterial accumulation in cord-like structures characteristic of extracellular growth [27], [28].

### Concluding remarks

Our work has shown that the automatic injector, coupled with COPAS analysis and sorting, provides an extremely powerful high-throughput pipeline for infecting and analyzing zebrafish embryos, and offers a new *in vivo* tool for rapidly testing the efficacy of large panels of molecules on the propagation of the pathogen studied. Gene-disruption tools, such as morpholinos, can be easily integrated into our set-up. Moreover, our results clearly demonstrate, for the first time, the potential of using fish larvae to investigate *M. tuberculosis* directly, and highlight the importance of the automatic injector in enabling a high biosafety-level study that would otherwise be technically extremely difficult to accomplish. Interestingly, we have recently demonstrated the applicability of our robotic injection system for the xenotransplantation of human tumour cell lines into zebrafish embryos (data not shown), showing its general relevance in the high-throughput study of diseases that benefit from the use of whole vertebrate organisms.

## Materials and Methods

### Automated injection system

A polycarbonate substrate featuring a honeycomb pattern of 1,024 hemi-spherical wells (1.3 mm diameter) was used to create a negative mould in flexible polydimethylsyloxane (PDMS, Sylgard 184, Dow Corning) using standard moulding techniques. A 1% agarose gel (Sphaero) was poured onto an agarose-coated glass plate and the PDMS mould was pressed to touch the glass. After gelling the mould was removed and the grid was placed in a leakage-free steel support (sized to a 96-well plate) (**Figure 4B**).

The embryo grid was placed in a motorized stage (MTmot 200×100 MR, Märzhäuser) connected to a controller (Tango, Märzhäuser).

A motorized micro-manipulator (Injectman II, Eppendorf) was adjusted to a vertical position above the stage, and connected to a pump (Femtojet Express, Eppendorf) featuring an external compressor (lubricated compressor, model 3–4, JUN-AIR).

A firewire camera (DFK41BF02.H, The Imaging Source) equipped with a 4× macro lens (MR4/O, The Imaging Source) was placed beneath the stage for imaging.

All components were connected to the controlling computer (Ubuntu AMD64). A multi-threaded control program was written in Python, using PySerial and wxPython. Coriander software (http://damien.douxchamps.net/ieee1394/coriander) was used for imaging.

The camera height was adjusted to focus on the top plane of the agarose grid, and a grid calibration was performed.

The grid was removed for loading with embryos (**Figure 4C**). The injection needle (pulled borosilicate glass capillary, Harvard Apparatus) was placed in the Injectman and moved to the central focal position. The x and y coordinates were stored and the needle was elevated to replace the filled grid.

The injection height was calibrated using the first embryo by moving the needle downwards through the chorion until touching the yolk (400 µm above injection point).

### Bacterial culture and inoculum preparation

*M. marinum* strain E11 stably expressing mCherry (pSMT3-mCherry vector) [31] was grown as previously described [32], in the presence of 50 µg/ml hygromycin. Injection inocula were prepared from glycerol stocks (frozen at OD600 = 0.75) by washing three times in sterile 0.05% Tween80/PBS solution (BD Difco), assessing optical density at 600 nm and resuspending in a 2% polyvinylpyrrolidone40 (PVP40) solution (CalBiochem) in PBS.

*M. marinum* Mma20 strains expressing, in addition to mCherry, mag49-GFP or gag7-GFP plasmids [23] were cultured in medium containing 20 µg/ml kanamycin and 50 µg/ml hygromycin.

*M. tuberculosis* strain H37Rv stably expressing mCherry was maintained in logarithmic phase at all times in 7H9 medium (BD Difco Middlebrook) containing 50 µg/ml hygromycin in a BSL-3 laboratory. Prior to injection, optical density at 600 nm was assessed, the bacteria were washed three times in sterile water and resuspended in 2% PVP40.

The number of CFU in each inoculum was verified by plating out serial dilutions and the injected inoculum in triplicate.

### Ethics statement

Zebrafish lines (wild-type, albino/fli1-egfp [33] and mpx-gfp [34]) were handled in compliance with the local animal welfare regulations and maintained according to standard protocols (zfin.org). The breeding of adult fish was approved by the local animal welfare committee (DEC) of the University of Leiden. All protocols adhered to the international guidelines specified by the EU Animal Protection Directive 86/609/EEC.

### Zebrafish infections

Infections including the Pu.1 morpholino [29] (1 mM, Gene Tools) were performed by yolk injection (1 nl) at the 1–2 cell stage, whereas all other injections of *M. marinum* (20–40 CFUs) or *M. tuberculosis* (100 CFUs) took place between 16 and 512 cells. Control embryos were injected with carrier solution.

After *M. marinum* infection, embryos were collected in 92×16 mm petri dishes (Sarstedt), with a maximum of 100 embryos per dish, and maintained at 28°C in egg water. At 3 dpi embryos were analyzed by the COPAS system (see below) and randomly split into two equal groups. One group was exposed to a combination of 200 µM Rifampicin (Sigma-Aldrich) and 2 mM Isoniazid (Sigma-Aldrich) for 3 days (exposure to the drugs achieved by adding compounds to egg water; antibiotics refreshed once daily) and the other was followed without treatment (water refreshed once daily). Uninjected controls were similarly split into treated and untreated groups to account for antibiotic effects. At 5 and 6 dpi, the different larva groups were analyzed by the COPAS system, and the bacterial load was assessed by the total red fluorescence detected.

After *M. tuberculosis* infection, embryos were collected in tanks containing 1 litre of egg water with a maximum of 300 embryos per tank, and maintained at 34°C. At 3 dpi embryos were randomly split into treated and untreated groups as described above. Twenty larvae per group were homogenized at 5 and 6 dpi and plated out to assess the number of live bacteria per larva. Batches of 40–100 larvae per group were fixed at 5 and 6 dpi for optical analyses.

### Immunohistochemistry

Larvae were fixed in 4% paraformaldehyde in PBS overnight at 4°C and immunolabeled using the L-plastin antibody as previously described [35].

### Microscopy

Fluorescence in embryos and larvae was observed using a Leica MZ16 FA fluorescence stereomicroscope equipped with LAS AF software (Leica Microsystems), a Leica DMI400 B confocal microscope equipped with LAS AF software (Leica Microsystems) and a Zeiss LSM5 Exciter/Axio Observer confocal microscope equipped with ZEN software (Carl Zeiss). The following objectives were used: Leica stereomicroscope PlanaP0 1x (**Figure 1A** **,** **2A and B** and **5B, C, E and F**); Leica confocal HCX PL Fluotar 40x/0.75 dry (**Figure 1B–I** **,** **2C–E** and **6B–D**); Zeiss confocal EC Plan-Neofluar 10x/0.30 dry (**Figure 6A**). Images were processed using the public domain program ImageJ (W. Rasband, ImageJ 1.42q, http://rsb.info.nih.gov/ij/).

### Complex Object Parametric Analyzer and Sorter (COPAS)

The COPAS™ XL (Union Biometrica) large particle sorter has been designed for the analysis, sorting and dispensing of objects up to 1.5 mm in diameter based on size, optical density and fluorescence intensity. It is equipped with 488 nm and 561 nm Solid State lasers, and mCherry is detected through a 615/24 Band-Pass filter.

The Profiler II Option simultaneously detects and analyzes up to 8,000 data points per object for each of the channels of extinction and fluorescence, and includes advanced imaging options. The resulting profiles can be used to set sorting parameters.

The COPAS parameters used were as follows: optical density threshold (extinction)  = 390 mV (COPAS value: 20); minimum time of flight  = 280 µs (COPAS value: 700); red photomultiplier tube (PMT) voltage  = 450 V; green PMT voltage  = 0 V; yellow PMT voltage  = 0 V.

### Statistics

The effect of drug treatment on *M. marinum* mCherry fluorescence in zebrafish larvae was statistically analyzed using a 2-tailed T-test. The correlation between drug treatment and the presence or absence of *M. tuberculosis* was determined by a χ^2^-test.

## Supporting Information

Figure S1 **Embryo holder designs.** Possible designs of the agarose embryo holder, demonstrating that the hemi-spherical alternative provides the largest injection target volume, taking embryo size variability into account (indicated by arrows). Three embryo sizes are depicted, where the embryo designated by “100” is of average size.
(TIF)

Figure S2 **Combinatorial treatment of** ***M. tuberculosis*** **-infected larvae.** Effect of combinatorial treatment on presence or absence of bacteria in 6 day-old larvae from two independent experiments.
(TIF)

Table S1 **Difference in mCherry-labelled** ***M. marinum*** **load between treated and untreated groups.** Both experiments indicate a highly significant reduction of bacterial numbers (measured by COPAS) as a direct result of treatment with first-line anti-TB drugs, as determined by a 2-tailed T-test.
(DOC)

Video S1 **Sample of automated injection of** ***M. marinum*** **into the yolk of early-stage zebrafish embryos.** The left panel allows a side-view of the injection process, whereas the right panel allows the visualization of the inoculum as it is injected into the yolk (camera located beneath the embryo). The video runs at normal speed.
(MP4)
