# Supplementary figures and images for: A High-Throughput Screen for Tuberculosis Progression
Source: PLoS One. 2011 Feb 16;6(2):e16779. doi: 10.1371/journal.pone.0016779 (PMC3040195; doi:10.1371/journal.pone.0016779)

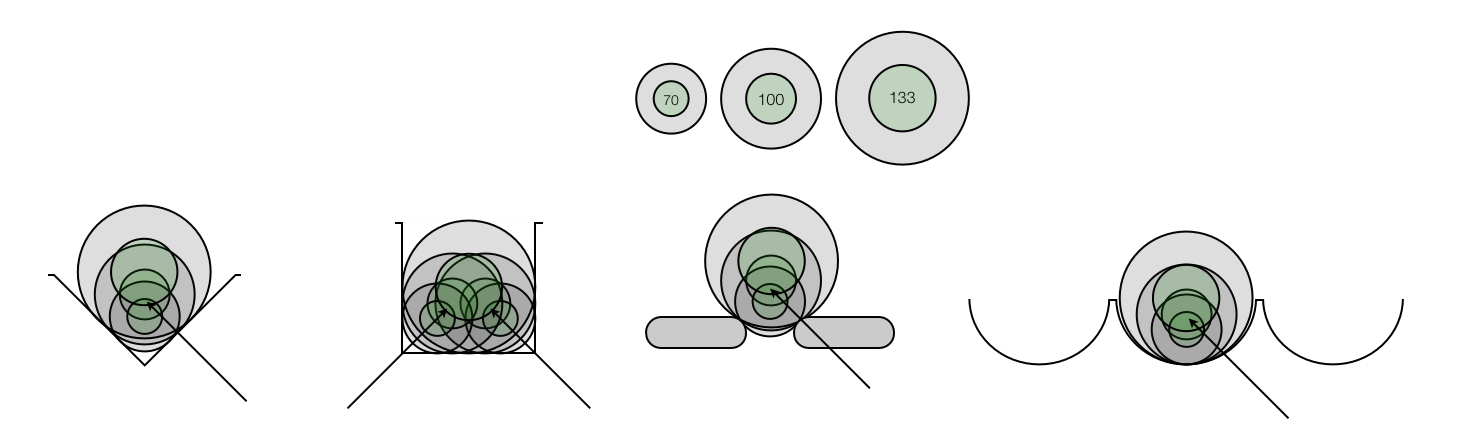

Supplement: Figure S1 — Embryo holder designs. Possible designs of the agarose embryo holder, demonstrating that the hemi-spherical alternative provides the largest injection target volume, taking embryo size variability into account (indicated by arrows). Three embryo sizes are depicted, where the embryo designated by “100” is of average size. (TIF) [file pone.0016779.s001.tif]

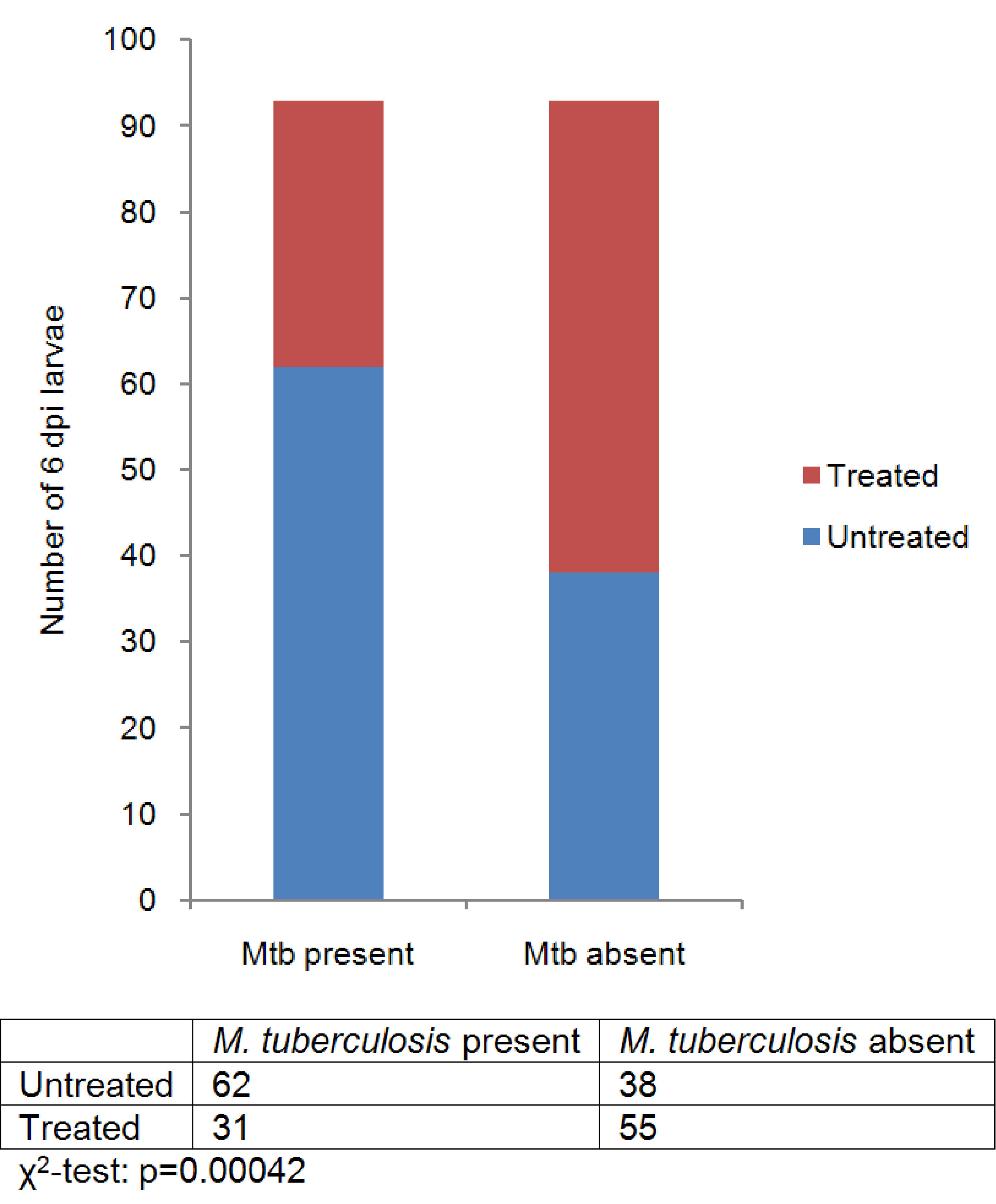

Supplement: Figure S2 — Combinatorial treatment of M. tuberculosis -infected larvae. Effect of combinatorial treatment on presence or absence of bacteria in 6 day-old larvae from two independent experiments. (TIF) [file pone.0016779.s002.tif]
