# Supplementary material for: A High-Throughput Screen for Tuberculosis Progression
Source: PLoS One. 2011 Feb 16;6(2):e16779. doi: 10.1371/journal.pone.0016779 (PMC3040195; doi:10.1371/journal.pone.0016779)
Supplement: Table S1 — Difference in mCherry-labelled M. marinum load between treated and untreated groups. Both experiments indicate a highly significant reduction of bacterial numbers (measured by COPAS) as a direct result of treatment with first-line anti-TB drugs, as determined by a 2-tailed T-test. (DOC) [file pone.0016779.s003.doc]

Supplementary Table

**Table S1. Difference in mCherry-labelled *M. marinum* load between treated and untreated groups**

| Number of embryos | Experiment number | Days post-infection | Exposure to treatment | Average red signal per embryo (mV) | Standard error of the mean | T-test |
| --- | --- | --- | --- | --- | --- | --- |
| 687 | 1 | 3 | n/a | 14.4 | 1.0 | n/a |
| 592 | 2 | 3 | n/a | 7.9 | 0.8 |
| 293 | 1 | 5 | Treated | 39.5 | 2.0 | 4.8E-22 |
| 274 | 1 | 5 | Untreated | 108.7 | 6.8 |
| 250 | 2 | 5 | Treated | 34.6 | 2.1 | 1.5E-19 |
| 275 | 2 | 5 | Untreated | 85.8 | 4.8 |
| 110 | 1 | 6 | Treated | 57.5 | 3.9 | 8.6E-9 |
| 127 | 1 | 6 | Untreated | 152.4 | 14.4 |
| 209 | 2 | 6 | Treated | 37.8 | 2.3 | 7.2E-30 |
| 179 | 2 | 6 | Untreated | 179.2 | 12.0 |
